# Supplementary material for: Leveraging current steering and the biophysics of spike generation for cellular-resolution electrical stimulation of neurons
Source: Cell Rep. Author manuscript; Available in PMC 2026 Apr 9. (PMC13065362; doi:10.1016/j.celrep.2025.116917)
Supplement: 1 [file NIHMS2158745-supplement-1.pdf]

Cell Reports, Volume *45*

Supplemental information

Leveraging current steering and the biophysics of spike generation for cellular-resolution electrical stimulation of neurons

Praful K. Vasireddy, Ramandeep S. Vilkhu, Amrith Lotlikar, Jeff B. Brown, A.J. Phillips, Alex R. Gogliettino, Madeline R. Hays, Claire Baum, Ethan J. Kato, Aviv Sharon, Pawel Hottowy, Alexander Sher, Alan M. Litke, Subhasish Mitra, Nishal P. Shah, and E.J. Chichilnisky

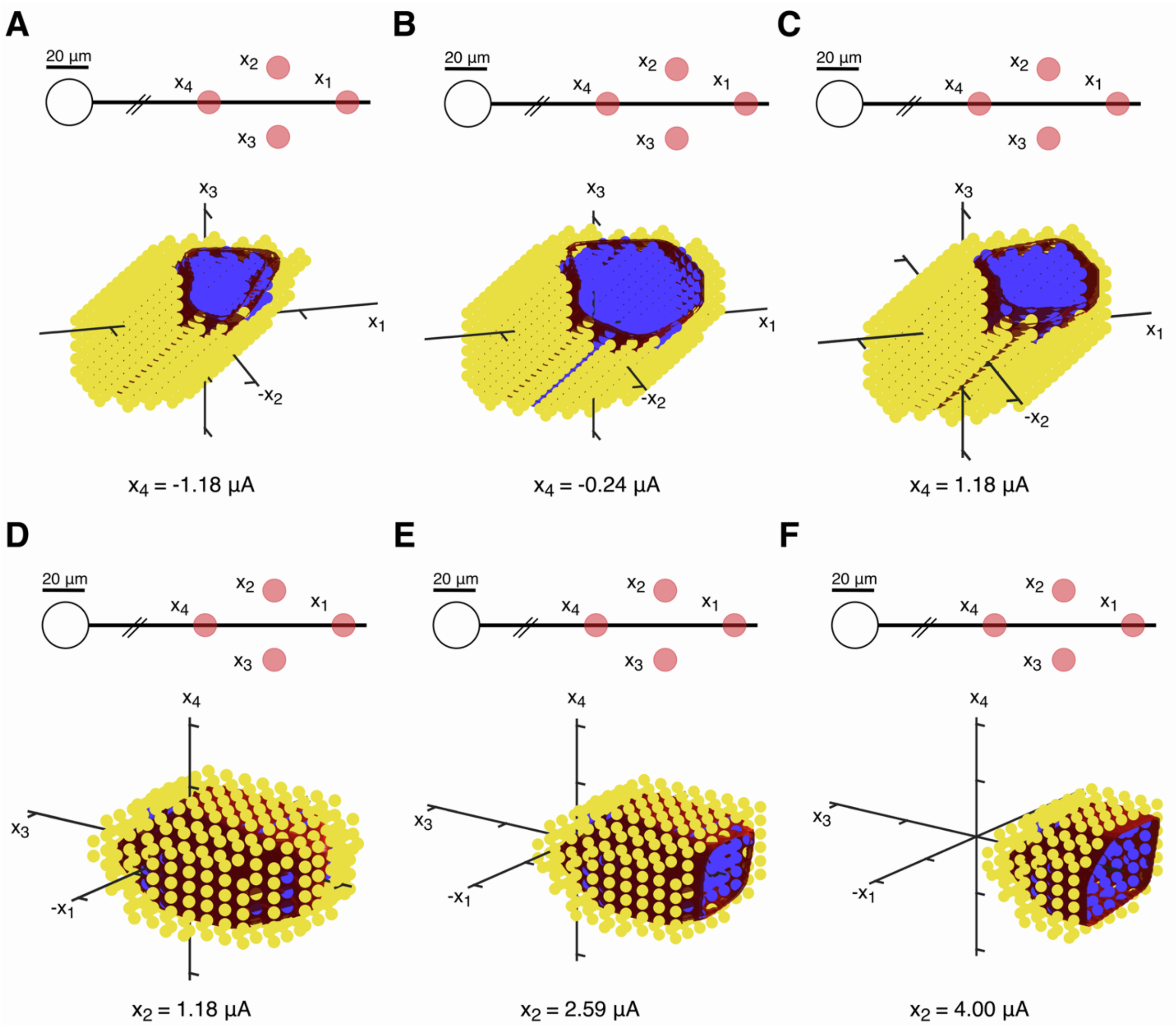

Figure S1. Extension of linear-OR model fitting to four-electrode stimulation responses in biophysical simulations

Nonlinear four-electrode stimulation biophysical simulation data with linear-OR model 50% surface for $m = 10$ activation sites. The linear-OR model fit had a McFadden pseudo-R^2 of 0.86, indicating an excellent fit (see Methods). Slices with freely varying amplitudes on three electrodes and fixed fourth electrode amplitude are shown. (A)—(C) Stimulation responses along three-electrode slices with fixed amplitude fourth electrode along the axon, for various fixed amplitude levels on the fourth electrode. (D)—(F) Stimulation responses along three-electrode slices with fixed amplitude fourth electrode transversely offset from the axon, for various fixed amplitude levels on the fourth electrode.

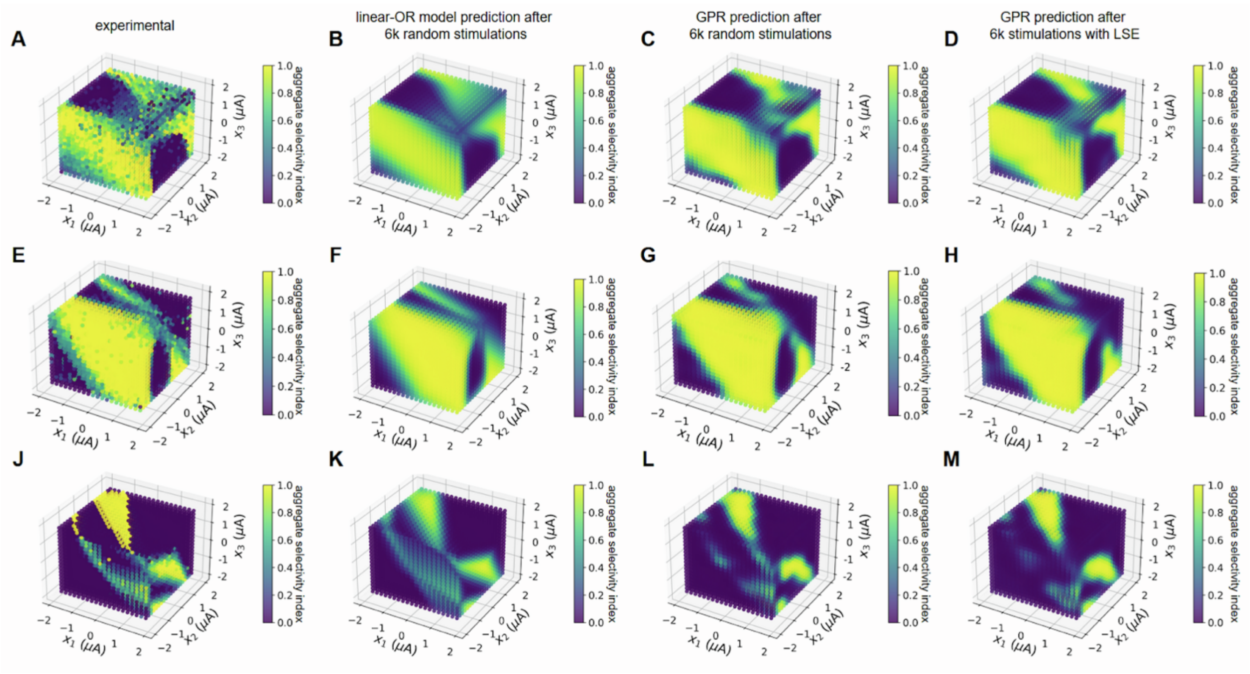

Figure S2. Gaussian process regression (GPR) and linear-OR model for estimation of aggregate selectivity profiles

First column shows experimentally measured aggregate selectivity profiles from full data (160,000 stimulations), second column shows linear-OR model prediction after fitting to subsampled data from each cell and computing the aggregate selectivity index, third column shows GPR mean prediction after performing 6,000 random calibrating stimulations, and fourth column shows GPR mean prediction after performing 6,000 calibrating stimulations guided by the GPR+LSE sampling procedure. Each row represents a distinct electrode triplet from a particular retinal preparation. (A)—(D) Macaque peripheral RGCs (2 cells aggregated). (E)—(H) Macaque central RGCs (5 cells aggregated). (J)—(M) Macaque peripheral RGCs (4 cells aggregated).
